# Theory of antiskyrmions in magnets

**DOI:** 10.1038/ncomms10542

**Published:** 2016-01-29

**Authors:** Wataru Koshibae, Naoto Nagaosa

**Affiliations:** 1 RIKEN Center for Emergent Matter Science (CEMS), 2-1 Hirosawa, Wako, Saitama 351-0198, Japan; 2 Department of Applied Physics, University of Tokyo, 7-3-1, Hongo, Bunkyo-ku, Tokyo 113-8656, Japan

## Abstract

Skyrmions and antiskyrmions are swirling topological magnetic textures realized as emergent particles in magnets. A skyrmion is stabilized by the Dzyaloshinskii–Moriya interaction in chiral magnets and/or a dipolar interaction in thin film magnets, which prefer the twist of the magnetic moments. Here we show by a numerical simulation of the Landau–Lifshitz–Gilbert equation that pairs of skyrmions and antiskyrmions are created from the helix state as the magnetic field is increased. Antiskyrmions are unstable and disappear immediately in chiral magnets, whereas they are metastable and survive in dipolar magnets. The collision between a skyrmion and an antiskyrmion in a dipolar magnet is also studied. It is found that the collision depends on their relative direction, and the pair annihilation occurs in some cases and only the antiskyrmion is destroyed in the other cases. These results indicate that the antiskyrmion offers a unique opportunity to study particles and antiparticles in condensed-matter systems.

## Abstract (web summary)

Skyrmions are swirling topological magnetic textures that behave as if they were particles. Here, the authors present numerical simulations that describe the creation and destruction of these spin vortices in both chiral and dipolar magnets, and show what happens when skyrmions and antiskyrmions collide.

## Graphical abstract

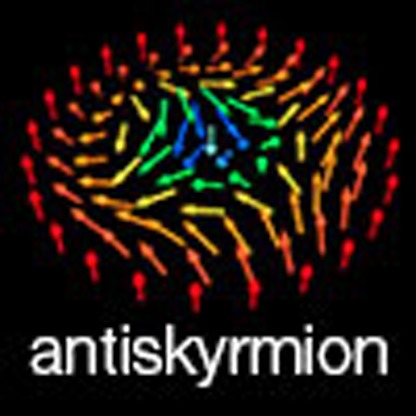

A particle in field theory is distinct from that in classical mechanics. It is regarded as the lump of the field configuration with higher energy and momentum compared with the ground state. Therefore, it can be created and annihilated, which usually occurs through interactions with the other particles. The most typical process is the pair creation and annihilation of a particle and its antiparticle. An antiparticle is usually characterized by the same mass and opposite charge to those of the particle. It is noteworthy that creation and annihilation are usually nonlinear processes, and its dynamics is related to the broad class of phenomena including nonlinear optics^1^ and cold atoms^2^. In magnets, the magnetic textures often behave as emergent ‘particles' made from many magnetic moments and the topological winding number has an important role there^3^. Among them, skyrmions^4^ are a swirling magnetic texture recently found in chiral magnets and also identified in dipolar magnets (magnetic bubbles), characterized by a topological integer called the skyrmion number *N*_sk_, attracts intensive interest both theoretically and experimentally^5^. The skyrmion number *N*_sk_ counts the number of times that the mapping **n**_**r**_ from the two-dimensional real-space coordinates **r** to the unit vector **n** along the magnetic moment wraps the unit sphere as given explicitly by




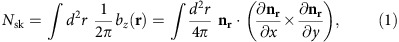




where *b*_*z*_(**r**) is the emergent magnetic field corresponding to the solid angle subtended by the magnetic moments. Skyrmion structure is parameterized as




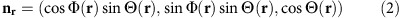




with




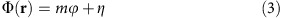







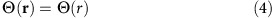




where **r**=(*r* cos *ϕ*, *r* sin *ϕ*). Assuming **n**=(0, 0, +1) for *r*→∞ (Θ(*r*→∞)=0) and **n**=(0, 0, −1) for *r*=0 (Θ(*r*=0)=*π*), the skyrmion number *N*_sk_ is determined by the vorticity *m* as *N*_sk_=−*m*. Here, *η* determines the helicity but is irrelevant to *N*_sk_. It is noteworthy that *N*_sk_ reverses its sign when all the directions of the magnetic moment are reversed, that is, **n**→−**n**, and this configuration can be called an ‘antiskyrmion'. However, the ‘antiskyrmion' in this study means the magnetic structure with *m*=−1 and *N*_sk_=+1 with the boundary condition **n**=(0, 0, 1) at *r*→∞.

Skyrmions are observed in several magnetic systems. A key ingredient is the Dzyaloshinskii–Moriya (DM) spin–orbit interaction^6^,^7^ allowed in the non-centrosymmetric magnetic crystals, namely the chiral magnets^8^,^9^,^10^,^11^,^12^,^13^,^14^,^15^,^16^,^17^,^18^,^19^,^20^,^21^,^22^. The other is the dipolar interaction^23^,^24^,^25^,^26^,^27^. The last one is the frustrated spin exchange interaction^28^. The size of the skyrmion with frustrated exchange interaction is of atomic size and depends on the geometry of the lattice. Therefore, we focus on the former two interactions, which give large size skyrmions and can be formulated in the continuum approximation. The important difference between the DM and dipolar interactions is that the former prefers a unique value of the helicity *η*, while the latter prefers two values of *η*. Therefore, skymions driven by dipolar interactions show much richer structures due to the helicity degrees of freedom as reported in ref. 25. In addition, it is noteworthy that for the antiskyrmion, the degree of freedom *η* plays a different role, that is, by a 90° rotation of the whole system, the magnetic texture of the antiskyrmion with *η*=−*π*/2 coincides with that of *η*=+*π*/2 for the same Θ(*r*). Therefore, *η* is not important for the single isolated antiskyrmion. This is in sharp contrast to the case of a skyrmion where the internal magnetic structure is different for different *η*'s (ref. 5).

The evolution in topological magnetic textures have been studied by several authors^29^,^30^,^31^. In the pioneering work by Cooper^29^, a ferromagnet without the DM or dipolar interaction was studied and it was found that the spin wave turned into a skyrmion-antiskyrmion pair as the momentum for the spin wave excitation is increased. In the present study, on the other hand, the DM or dipolar interaction breaks the translational symmetry in the ground states as shown both theoretically^5^,^23^,^24^,^34^ and experimentally^15^,^25^. Namely, the single-*q* helix state changes into the skyrmion crystal state (SkX) as the magnetic field increases. This is a topological phase transition in the magnetic texture; that is, the skyrmion number *N*_sk_ is zero in the single-*q* helix state but *N*_sk_≠0 in the SkX. As the processes changing the skyrmion number require the discontinuous magnetic configurations and hence it has a large energy barrier^3^,^5^, the dynamics of the magnetic structures with the increased magnetic field is a highly non-trivial issue, which we address below.

In the following, we study the skyrmions and antiskyrmions in chiral and dipolar magnets at zero temperature by numerically solving the Landau–Lifshitz–Gilbert equation to explore their creation, stability, interaction, dynamics and collisions.

## Results

### Model and simulation

**Figure 1:**
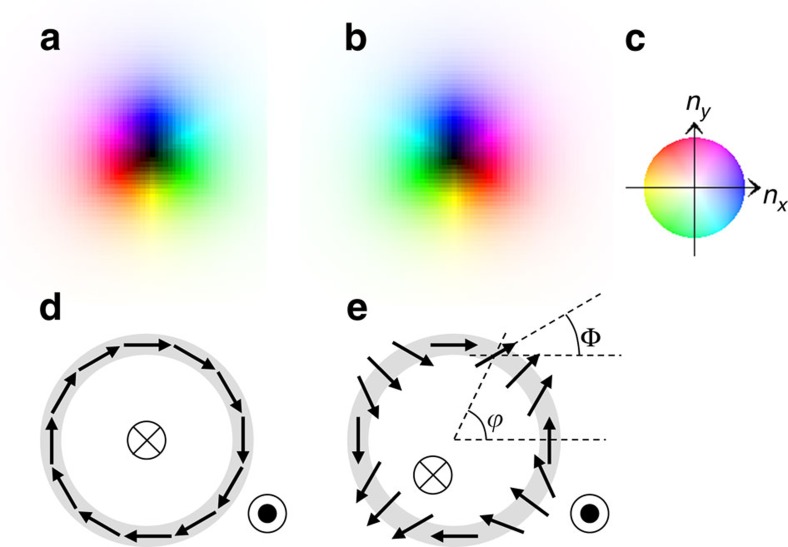
Skyrmion and antiskyrmion. (**a**,**b**) Skyrmion (with {*N*_sk_=−1, *m*=1, *η*=−*π*/2} in equations (1, 2, 3, 4)) and antiskyrmion (with {*N*_*sk*_=+1, *m*=−1, *η*=+*π*/2}) structures, respectively, using the colour coding (**c**) for the magnetic moment **n**_**r**_. For example, blue is used for *n*_*x*_>0 and *n*_*y*_=0. The brightness of the colour indicates the *z*-component *n*_*z*_ of **n**, that is, the bright (dark) colour is for positive (negative) large *n*_*z*_ and hence white (black) corresponds to the north pole *n*_*z*_=1 (south pole *n*_*z*_=−1). (**d**,**e**) The in-plane magnetic structures of the skyrmion and antiskyrmion for a constant *r*=|**r**|, respectively, and the in-plane direction of **n**_**r**_ is specified by the angle Φ at *ϕ*. The symbols 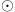 and ⊗ indicate the direction of the out-of-plane component *n*_*z*_≈+1 and *n*_*z*_≈−1, respectively.

The model Hamiltonian for the chiral magnets defined on the two-dimensional square lattice is given by




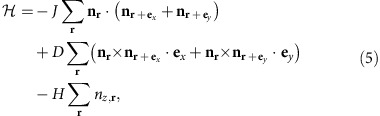




where **e**_*x*_ and **e**_*y*_ are the unit vectors in the *x* and *y* directions, respectively, and we take the lattice constant as the unit of length. The combination of the exchange interaction *J* and DM interaction *D* produces the single-*q* helix state with *q*=*D*/*J* under zero magnetic field. Usually, 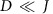 and the helix period *ξ*=2*π*/*q*=2*π*(*J*/*D*) is much longer than the lattice constant, and hence the continuum approximation is justified. Long *ξ* indicates the small energy scale *E*_1_∼*J*/*ξ*^2^ per magnetic moment, which is the stabilization energy density for the magnetic textures due to the DM interaction. Under an external magnetic field *H*, the Zeeman energy prefers the ferromagnetic state and the SkX appears in the intermediate *H* between the helix state for low *H* and the ferromagnetic state for high *H*^32^,^33^. This is because SkX can gain energy both from the DM interaction and Zeeman interactions. As expected, the natural scale of the magnetic field is *E*_1_ defined above and SkX appears when *H*∼*E*_1_. For *D*>0, the DM interaction prefers *η*=−*π*/2 for the skyrmion (the magnetic texture is shown in Fig. 1a). In the case of antiskyrmion, however, the energy gain (cost) occurs depending on *ϕ*. Figure 1b is a schematic representation of the antiskyrmion described in equations (2, 3, 4) with *m*=−1 and *η*=+*π*/2. In this magnetic texture, the winding of **n**_**r**_ along the radial direction costs (gains) energy by DM interaction for *ϕ*=0, *π* (*ϕ*=*π*/2, 3*π*/2). Therefore, the magnetic texture distorts from those described by equations (2, 3, 4) and Θ also depends on *ϕ* to reduce the radius of the antiskyrmion for *ϕ*=0, *π* and energy cost.

Topological protection means that as long as the low-energy phenomena of the order of 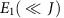 are concerned, slowly varying (continuous) magnetic structures are relevant and hence *N*_sk_ is conserved. In other words, the high-energy magnetic structures with the discontinuous change cost energy of the order of 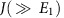 and hence is suppressed. Therefore, it is an important issue how this topological protection works in the dynamics of chiral magnets.

As for the dipolar magnet, the Hamiltonian reads




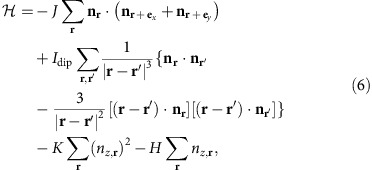




where *I*_dip_ and *K* represent the dipole interaction and the uniaxial anisotropy, respectively. The phase diagram under magnetic field is similar to that of DM magnet^34^. The evolution in magnetic texture is also experimentally observed^25^, that is, the helix ground state is seen at zero magnetic field and the skyrmionic state emerges as the magnetic field is increased. The wavenumber *q* of the helix structure is given by *q*≅(*I*_*dip*_/*J*)^1/3^ in this case and corresponding energy scale is 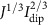. The magnetic dipolar energy is represented by the Coulomb-like interaction between the magnetic charge *ρ*_*m*_=−div **n**. As div **n**=0 for skyrmions with *η*=+*π*/2 and −*π*/2, these two skyrmion states are degenerate in this case. For the antiskyrmion, on the other hand, the energy cost occurs, that is, for {*N*_sk_=+1, *m*=−1, *η*=+*π*/2} in equations (2, 3, 4) and in Fig. 1b, we find *ρ*_*m*_=−[cos Θ(*r*)∂_*r*_Θ(*r*)−(1/*r*) sin Θ(*r*)] sin 2*ϕ*, so that the antiskyrmion state loses the energy gain due to the interaction of the magnetic dipoles. This remains qualitatively true when we consider the *ϕ* dependence of Θ, which is neglected in equations (2, 3, 4).

The Landau-Lifshitz-Gilbert equation is given by:




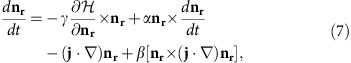




where *α* is the Gilbert damping constant. The last two terms in equation (7) represent the spin–transfer–torque effect due to the magnetically polarized electric current **j** with the coefficient of the non-adiabatic effect *β*. We use 1/(*γJ*) for the unit of time *t*. Typically, *J*∼10^−3^ eV and the unit 1/(*γJ*) becomes ∼0.7 ps for *γ*=*g*_*s*_*μ*_*B*_/*ℏ* (*g*_*s*_, electron spin *g*-factor and *μ*_B_, Bohr magneton). The unit of the electric current density *j*=|**j**| is 2*eγJ*/*pa*^2^ and is typically ∼1.0 × 10^13^ Am^−2^ for the polarization of magnet *P*=0.2 and the lattice constant *a*=5 Å.

### Pair creation of skyrmion and antiskyrmion in chiral magnet

First, we consider the creation of antiskyrmion in the chiral magnet described by the Hamiltonian equation (5), that is, the evolution of the magnetic texture starting from the helix ground state with increasing magnetic field. (see Supplementary Note 1 for computational details.)

**Figure 2:**
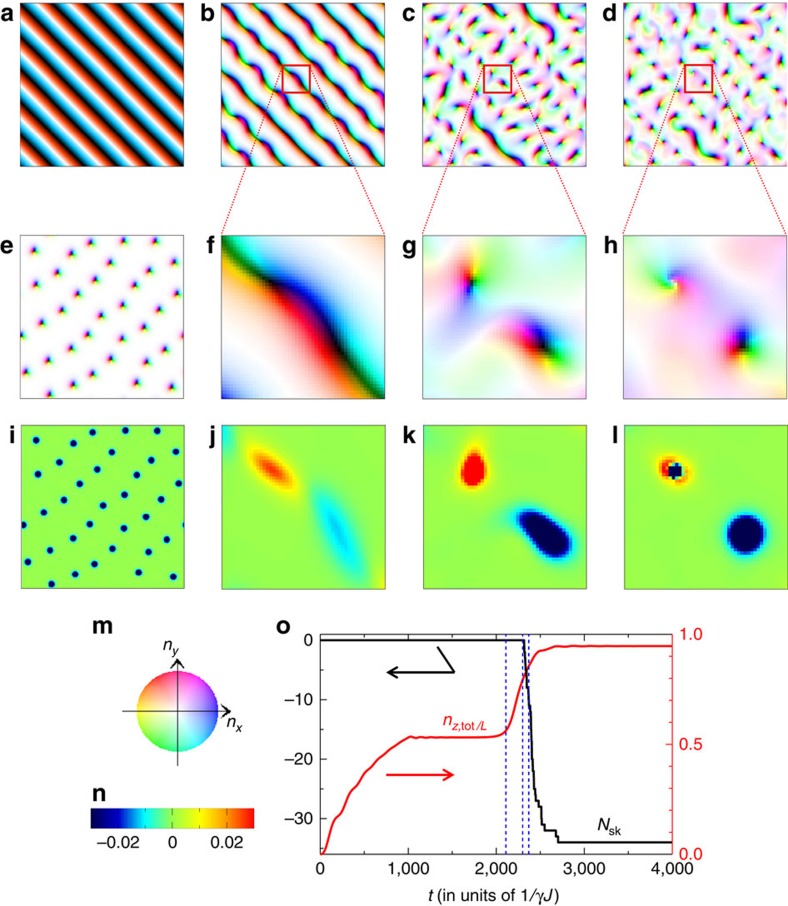
Magnetization process of a chiral magnet. (**a**)→(**b**)→(**c**)→(**d**)→(**e**) The time evolution in the magnetic texture. The colour code (**m**) is the same as in Fig. 1. (**a**) Initial state. The system size is *L*=300 × 300. (**b**–**e**) The snapshots of magnetic structure at *t*=2,110, 2,300, 2,369 and 4,000 in units of 1/(*γJ*), respectively. The close-ups of the regions enclosed by the red squares (50 × 50) in **b**–**d** are shown in **f**–**h**, respectively. (**i**–**l**) The spatial distribution of the solid angle formed by four magnetic moments on plaquette, 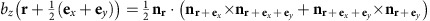 for the magnetic textures shown in **e**–**h**, respectively. The scale of *b*_*z*_ is shown in **n**. Blue indicates the skyrmion, while red indicates the antiskyrmion. (**o**) Time dependence of skyrmion number *N*_sk_ (black) and *z*-component of total magnetic moment 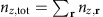 divided by system size *L* (red). The blue vertical broken lines indicates *t*=2,110, 2,300 and 2,369, respectively. See Supplementary Movies 1–4.

Figure 2 shows the magnetization process of a finite size *L*=300 × 300 system with periodic boundary condition for a parameter set {*D*/*J*=0.15, *α*=0.1}. We use a ‘switching-on' procedure to apply the magnetic field *H*. For 0<*t*<*t*_0_, the applied magnetic field *H* is an increasing function of *t*, that is, *H*=*H*_0_/*t*_0_ × *t* and *H*=*H*_0_ for *t*>*t*_0_. The parameters, *H*_0_/*J*=0.04 and *t*_0_=1,000 are used here. Figure 2a–e shows the time evolution of the magnetic structure. The close-ups of the regions enclosed by the red squares (50 × 50) in Fig. 2b–d are shown in Fig. 2f–h, respectively. The spatial distribution of the solid angle formed by four magnetic moments on plaquette, 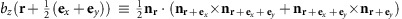 for Fig. 2e–h are shown in Fig. 2i–l, respectively. It has been known that *b*_*z*_ has the physical meaning of the emergent magnetic field acting on the conduction electrons coupled to the magnetic moments^35^,^36^,^37^. The blue colour means the negative *b*_*z*_ corresponding to the skyrmion, while the red colour means the positive *b*_*z*_ and antiskyrmion. It is noteworthy that 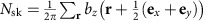 and its time dependence is shown by the black line, and 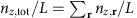 by the red curve in Fig. 2m. The initial state shown in Fig. 2a is the ground state for *H*=0. More explicitly, 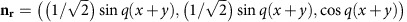 for **r**=(*x*, *y*), where *q*=|**q**|∼*D*/*J* and **q**=(*π*/30, *π*/30) in this simulation (see Supplementary Note 1). In the single-**q** helix state, because the magnetic moments are winding in the plane perpendicular to **q**, *b*_*z*_=0 everywhere. Starting from this initial state, the magnetic field is switched on as described above, so that this system shows a dynamics of magnetization process. It is noteworthy that the critical magnetic field *H*_*c*_^33^ separating the skyrmion crystal and the ferromagnetic ground states is *H*_*c*_/*J*≈0.0175 with *D*/*J*=0.15 used in the simulation. Therefore, the ground state is the ferromagnetic one at *H*/*J*=0.04, whereas the SkX is a metastable state. After the shrinkage of the negative *n*_*z*_ region, the helix magnetic texture changes into the wavy stripe pattern as shown in Fig. 2b and the pair creation of skyrmion–antiskyrmion begins. The dark colour area *n*_*z*,**r**_≃−1 is thin and the wavy stripe pattern shows a precursory instability to break. It is noteworthy that this instability occurs at wavevector ∼*D*/*J* perpendicular to **q**, due to the DM interaction. Figure 2f is a close-up of the magnetic structure of Fig. 2b inside the red square, that is, the magnetic moments with *n*_*z*,**r**_≃−1 twist themselves upward in positive *n*_*x*_ and *n*_*y*_ direction (blue region) at the centre and in the opposite direction (yellow region) in the outsides (see also the colour code in Fig. 2m). It is interesting to note that this initial instability with a long wavelength is apparently similar to the ‘snake instability', which has been studied for the solitary excitation in nonlinear dispersive media^1^,^2^. Along with the change in magnetic texture, a spatial inhomogeneity in *b*_*z*_ appears as shown in Fig. 2j; yet, the spatial summation of *b*_*z*_, that is, *N*_sk_ is still zero (see the black line in Fig. 2m at *t*=2,110). The larger in-plane components of the magnetic moments give the larger torque for the magnetic moment under the finite magnetic field *H*, so that the twisting motion is accelerated. Consequently, a large number of magnetic moments saturate at *n*_*z*,**r**_≃+1; however, the magnetic moments stay at *n*_*z*,**r**_≃−1 between the blue and yellow regions, which will form the cores of skyrmion and antiskyrmion as seen in Fig. 2j. After this change, skyrmion–antiskyrmion pair appears (at *t*=2,300) as shown in Fig. 2c,g,k. This skyrmion–antiskyrmion creation process is seen over the whole system. It is noteworthy that for the configuration shown in Fig. 2c,g,k at *t*=2,300, the skyrmion number *N*_sk_ is still zero. This is because the numbers of skyrmions and antiskyrmions are equal. Later on, *N*_sk_ decreases. In this stage, the individual skyrmion or antiskyrmion disappears. Figure 2h,l shows the magnetic textures at the moment of antiskyrmion annihilation. There are no pair annihilation of skyrmion and antiskyrmion observed during this numerical simulation. Eventually, all the antiskyrmions and some of skyrmions disappear, which results in the metastable skyrmion crystal-like state (see Fig. 2e,i), whereafter the magnetic structure hardly change, although the ground state for *H*/*J*=0.04 is the perfect ferromagnetic state. It is noted here that the whole process occurs within the very short time scale of the order of nanosecond. Namely, it is very difficult to observe the antiskyrmions in chiral magnets in terms of the slow probe such as Lorentz transmission electron microscopy. It is noted that *N*_sk_ is always an integer in the whole time period. We also studied the cases of different speed for the change in the magnetic field, but the results are similar to those presented above.

As seen in Fig. 2(c)→(d), the antiskyrmion annihilation occurs by the contraction of the domain of *n*_*z*_≈−1 where the magnetic moment changes the direction within a few lattice spacing. At this stage, the topology of the magnetic texture is no longer well defined and hence the topological transition occurs along the time evolution to reach the ‘flat' magnetic texture. In the chiral magnet, the antiskyrmion always involves the winding of the magnetic moments competing with the DM interaction and this gives a driving force to shrink the antiskyrmion to a few lattice spacings.

### Pair creation of skyrmion and antiskyrmion in dipolar magnet

In the dipolar magnet, the skyrmion and antiskyrmion have been studied in the context of so-called normal/hard magnetic bubbles (for example, see refs 23, 24, 25, 34). Because of the out-of-plane uniaxial anisotropy, one can consider the in-plane magnetic moment along the circular domain wall characterized by Φ(*ϕ*) as **n**=(cos Φ, sin Φ). The effective Hamiltonian for this variable is given by




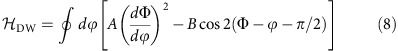




where *A* comes from the exchange interaction and *B* from the dipolar interaction. The angle Φ=*ϕ*±*π*/2 describes the usual Bloch wall configuration and hence a skyrmion, whereas the antiskyrmion corresponds to the configuration of four Bloch lines (BLs) with Néel wall, that is, the in-plane magnetic moments are (almost) along radial direction at the BLs (see Fig. 1e). This costs energy by the dipolar interaction, but the repulsive interaction between the BLs gives the stability of this configuration as long as the reduction of the domain wall radius is protected by the magnetic anisotropy^24^.

**Figure 3:**
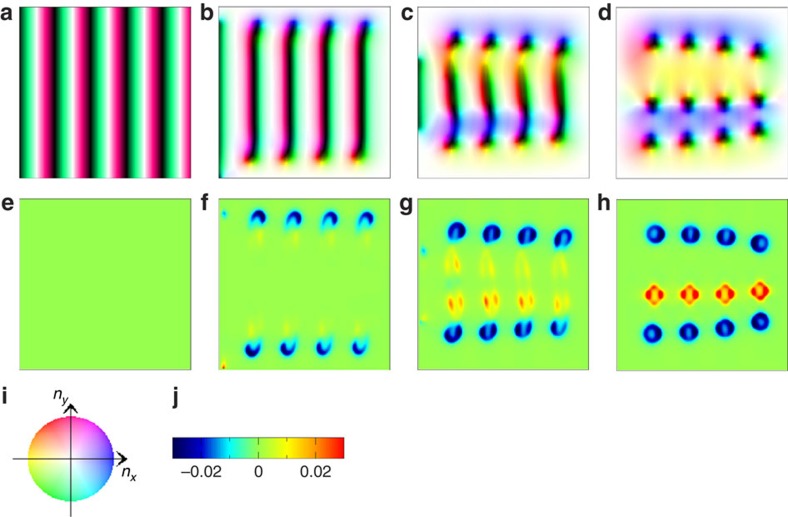
Pair creations of skyrmion–antiskyrmion for dipolar magnet. A parameter set {*L*=150 × 150, *I*_dip_/*J*=0.094, *K*/*J*=0.6, *H*/*J*=0.04, *α*=1.0} with open boundary condition (OBC) is used. (**a**) Initial state. (**b**–**d**) The snapshots of magnetic structure at *t*=200, 450 and 1,000 in units of 1/(*γJ*), respectively. The colour code (**i**) is the same as in Fig. 1. (**e**–**h**) The spatial distribution of *b*_*z*_. The scale of *b*_*z*_ is shown in **j**.

The pair creations of skyrmion–antiskyrmion in dipolar magnet is shown in Fig. 3. Because of the long-range nature of the dipolar interaction, we employ the open boundary condition for the square shaped sample. Here we start with a single-**q** helix state. Along the time evolution, Fig. 3(a)→(b), the helix structure begins to deform from the boundaries to form elongated skyrmions. In Fig. 3b, each half of the skyrmion number is distributed near the top and bottom parts. As time proceeds, each elongated skyrmion shows its continuous deformation (see Fig. 3(b)→(c)→(d)) and is pinched at two regions to form an antiskyrmion in the middle and, at the same time, each half-skyrmion develops into a whole skyrmion. Therefore, the total skyrmion number does not change. After this skyrmion–antiskyrmion pair formation, these particles are stable and do not disappear within the simulation time, which is in sharp contrast to the case of chiral magnet.

### Interaction between skyrmion and antiskyrmion

**Figure 4:**
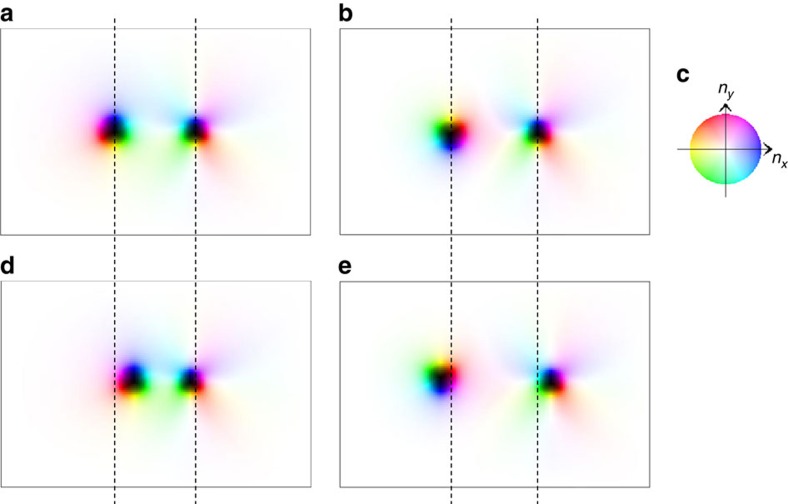
Interaction between skyrmion and antiskyrmion in dipolar magnet. A parameter set {*L*=150 × 100, *I*_dip_/*J*=0.092, *K*/*J*=0.6, *H*/*J*=0.04, *α*=0.01} with open boundary condition (OBC) is used. (**a**,**b**) The initial states with different arrangements in in-plane magnetic texture. The colour code is shown in **c**. (**d**,**e**) The results of time evolution at *t*=15,500 in units of 1/(*γJ*) from the initial states in **a** and **b**, respectively; **a** and **b** correspond to the initial conditions in Fig. 5a,i, respectively.

Having established the metastability of an antiskyrmion in dipolar magnets, we will study its interaction with a skyrmion. For this purpose, we first put one skyrmion and one antiskyrmion at rest and trace their time-evolution. Here we consider two cases (see Fig. 4a,b): The skyrmion with (i) *η*=−*π*/2 or (ii) *η*=+*π*/2 is put at left of the antiskyrmion with *η*=+*π*/2. In case (i), the alignment of the *n*_*y*_ component between the skyrmion and antiskyrmion is compatible with the ferromagnetic interaction *J* (the leading interaction of the Hamiltonian equation (6)) but not in case (ii). These two cases are exchanged by rotating the antiskyrmion or by changing the relative position of the skyrmion and antiskyrmion. Figure 4 shows the initial state and the snapshot of the magnetic texture at *t*=15,500 in units of 1/(*γJ*) for each case of (i) and (ii). It is seen that the two particles attract each other and approach to a shorter distance in Fig. 4a (case (i)), while they repel each other in Fig. 4b (case (ii)). These results indicate that the attractive interaction occurs between the skyrmion and antiskyrmion at least in some finite range of the distance in case (i), while the interaction is repulsive in case (ii). It is noteworthy here that the magnetic charge div **n**=0 for the skyrmion, although it is non-zero for the antiskyrmion, and hence there is no long-range interaction between them. In fact, the interaction seems to be short ranged in our simulation, although the sample size is still too small to conclude the range of the interaction convincingly. A clue to understand this interaction between a skyrmion and an antiskyrmion can be found in ref. 40, where the interaction between the two Bloch walls are discussed. Braun (ref. 40) analysed the twisted and untwisted pairs of *π*-Bloch walls and found the stability of the former compared with the latter against the annihilation by external magnetic field. This can be translated to the attractive (repulsive) interaction between the skyrmion and antiskyrmion in the configuration in Fig. 4a,d (Fig. 4b,e) when the twist of magnetic moments along the line connecting the centres of these structures is regarded as the pair of Bloch walls. We did not observe the pair annihilation of these two particles even in the case of (i). Therefore, we put the external current to drive the collision as follows.

### Skyrmion-antiskyrmion collision and pair annihilation

**Figure 5:**
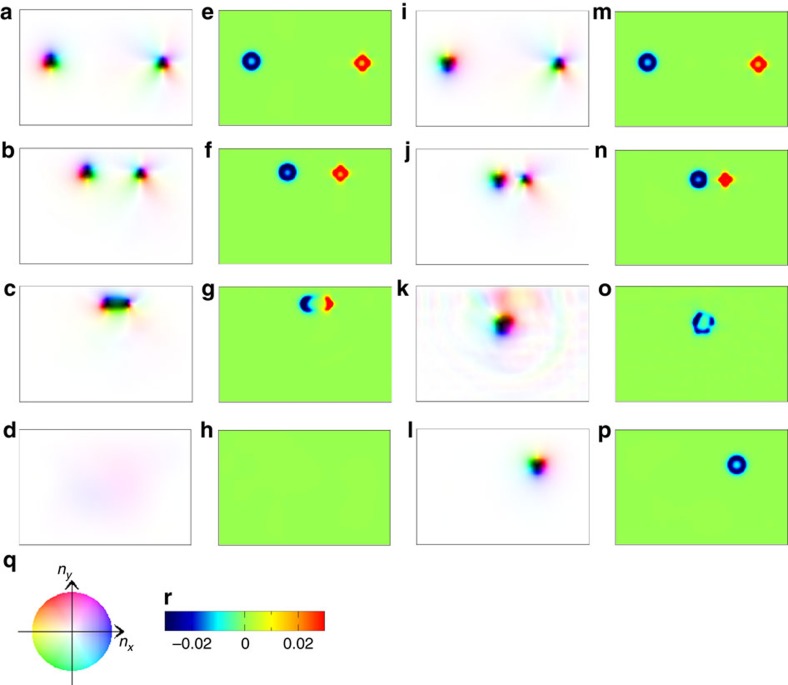
Skyrmion and antiskyrmion collision in dipolar magnet. A parameter set {*L*=150 × 100, *I*_dip_/*J*=0.092, *K*/*J*=0.6, *H*/*J*=0.04, *α*=0.01, *β*=0.0, *j*=0.001} with open boundary condition (OBC) is used. (**a**) The initial state at rest (case (i) in the text.) The colour code (**q**) is the same as in Fig. 1. (**b**–**d**) The snapshots of the time evolution under the electric current flowing from the upper to lower edges at *t*=6,000, 7,700 and 15,000 in units of 1/(*γJ*), respectively. (**e**–**h**) The spatial distribution of *b*_*z*_ for the magnetic textures shown in **a**–**d**, respectively. The scale of *b*_*z*_ is in **r**. (**i**–**p**) The results of **n**_**r**_ and *b*_*z*_ in case (ii) in text at *t*=0, 7,700, 8,400 and 15,000 in units of 1/(*γJ*), respectively. See Supplementary Movies 5 and 6.

The motion of the skyrmion and antiskyrmion under external current is the same in the absence of the potential from the boundary, except for a small skyrmion Hall effect, that is, their velocity is just given by that of the conduction electrons^38^,^39^,^40^,^41^. However, in the presence of the confining potential, it strongly depends on the skyrmion charge *N*_sk_. A representative example is the current-driven motion along the edge of the sample^42^,^43^,^44^,^45^. The motion along the edge driven by the perpendicular current to the edge is in opposite directions for the opposite sign of *N*_sk_ and its velocity is enhanced by the factor of *α*^−1^ compared with that of the free space^43^,^44^. We use this fact to collide a skyrmion and an antiskyrmion as shown in the snapshots of Fig. 5. Here, the electric current is applied in the −*y* direction. Therefore, the motion of the skyrmion and antiskyrmion is accelerated in +*y* direction and collide along the upper edge of the system. There are two cases (i) and (ii) as in Fig. 4. In the case (i), the skyrmion (*N*_sk_=−1) and antiskyrmion (*N*_sk_=+1) merge into a single composed magnetic texture with *N*_sk_=0, so-called type-II magnetic bubble (see Fig. 5(a)→(c) and Fig. 5(e)→(g)). This evolution in the magnetic texture occurs within a continuous deformation, because the skyrmion number is conserved in total. As the type-II magnetic bubble has *N*_sk_=0, this can disappear into the perfect ferromagnetic background (see Fig. 5(c)→(d) and Fig. 5(g)→(h)) within a continuous deformation in the magnetic texture depending on the parameters, external magnetic field and the magnetic anisotropy. This is the pair annihilation process of a skyrmion and antiskyrmion in the case (i).

On the other hand, in Fig. 5i–p for case (ii), although the skyrmion and antiskyrmions approach first by the current, the skyrmion survives after this collision, while the antiskyrmion is annihilated and the skyrmion number *N*_sk_ is changed by 1.

**Figure 6:**
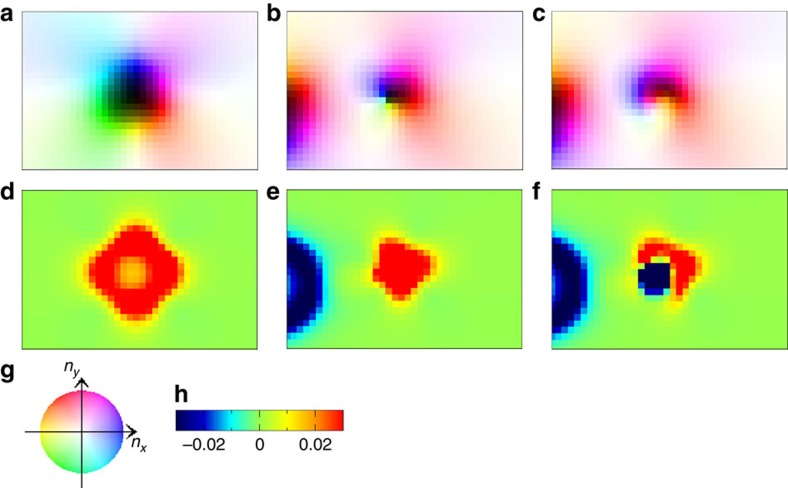
Details of annihilation processes of antiskyrmion. Snapshots during the process discussed in Fig. 5(i)→(j)→(k) and (m)→(n)→(o) are presented here. (**a**–**c**) The close-ups of the magnetic texture of antiskyrmion at *t*=0, 8,320 and 8,324, respectively. The area of size 37 × 25 around the antiskyrmion is zoomed up in the system *L*=150 × 100. The colour code (**g**) is the same as in Fig. 1. (**d**–**f**) The spatial distribution of *b*_*z*_ for the magnetic textures shown in **a**–**c**, respectively. The scale of *b*_*z*_ is shown in **h**.

Looking at these processes, the cases (i) and (ii), more carefully, the role of the BLs along the domain wall of antiskyrmion becomes evident. In an antiskyrmion (see Fig. 1e), there are four BLs. The two of them on the left side in the case (i) are annihilated to merge into the domain wall of the skyrmion, while the two on the right side remain to form a type-II bubble (see Fig. 5c) with the negative (blue) and positive (red) *b*_*z*_ on the left and right halves, respectively (see Fig. 5g). On the other hand, in case (ii), the two BLs on the left side annihilate pairwise to leave the region of Bloch walls consistent with that of neighbouring skyrmion. This results in the temporal type-II bubble turned from the antiskyrmion with the skyrmion kept intact. This is seen in Fig. 6, which shows the magnetic texture and the spatial distribution of *b*_*z*_ during the annihilation process of the antiskyrmion.

The results seen in Figs 5 and 6 indicate the difference in the stability of skyrmion and antiskyrmion, but the reaction of the skyrmion and antiskyrmion depends strongly on the direction of the antiskyrmion or the collision.

## Discussion

We have theoretically studied the antiskyrmion in chiral and dipolar magnets. It is metastable in dipolar magnet, while it is not in chiral magnet. Therefore, we expect the rich phenomena associated with antiskyrmions in dipolar magnets. Manipulation of the antiskyrmions by various method such as the optical generation of spin waves, current-driven motion and by strain will be an interesting direction to pursue experimentally. In addition, it has been shown theoretically that the hotspot by, for example, laser irradiation in the ferromagnetic state can create the antiskyrmions in dipolar magnets^26^,^46^,^47^. The antiskyrmion is strongly anisotropic in shape, that is, fourfold symmetric form, which produces angle-dependent phenomena. Already, we have shown that the interaction between a skyrmion and an antiskyrmion is found to be strongly angle dependent. In the collision process driven by the current, the pair annihilation occurs in the case of attractive direction, whereas only the antiskyrmion is destroyed in the repulsive direction. In addition, the interaction between the edge and an antiskyrmion is expected to be angle dependent. These rich physics associated with the antiskyrmions in dipolar magnets, which can be studied in the table-top experiment, will shed light on the birth and death of the topological particles and antiparticles.

## Additional information

**How to cite this article:** Koshibae, W. & Nagaosa, N. Theory of antiskyrmions in magnets. *Nat. Commun.* 7:10542 doi: 10.1038/ncomms10542 (2016).

## Supplementary Material

Supplementary Information Supplementary Note 1.

Supplementary Movie 1 Magnetic texture of the results in Fig. 2, for t=0~4000 in units of 1/γ*J.* The color code shown below the Fig. 2(a) is used.

Supplementary Movie 2 Spatial structure of bz for the results in Fig. 2, for t=0~4000 in units of 1/γ*J.* The color scale shown above the Fig. 2(i) is used.

Supplementary Movie 3 Magnetic texture in the focused area (indicated by red rectangles in Fig. 2(b)-(d)) of the results in Fig. 2, for t=2000~2800 in units of 1/γ*J.* The color code shown below the Fig. 2(a) is used.

Supplementary Movie 4 Spatial structure of bz in the focused area indicated by red rectangles in Fig. 2(b)-(d), for t=2000~2800 in units of 1/γ*J.* The color scale shown above the Fig. 2(i) is used.

Supplementary Movie 5 Magnetic texture of the skyrmion-antiskyrmion pair annihilation shown in Fig. 5, for t=0~15000 in units of 1/γ*J.* The color code shown below the Fig. 5(a) is used. The spin-transfer-torque effect along the upper edge of the system is due to the confining force at the edge (Refs.[43-45] in main text). This effect also appears for the interaction between the skyrmion-antiskyrmion: there exist an attractive interaction between them in this case, motion in +y direction appears for closer skyrmion-antiskyrmion pair and the resulting type-II magnetic bubble.

Supplementary Movie 6 Magnetic texture for the case that the skyrmion (antiskyrmion) survives (annihilates) pair annihilation shown in Fig. 5, for t=0~15000 in units of 1/γ*J.*. The color code shown below the Fig. 5(a) is used. In contrast to the case shown in "Supplementary Movie 5", the closer skyrmion-antiskyrmion pair moves in -y direction, because there exist a repulsive interaction between them.
