# Supplementary material for: Theory of antiskyrmions in magnets
Source: Nat Commun. 2016 Jan 29;7:10542. doi: 10.1038/ncomms10542 (PMC4740186; doi:10.1038/ncomms10542)
Supplement: Supplementary Information — Supplementary Note 1. [file ncomms10542-s1.pdf]

“Supplementary Note 1”

Computational details for the results in Fig. 2

In the calculations of the results presented in Fig. 2, we take the following procedure: Due to a finite size effect, a commensurate single-**q** helical state with a wave vector **q**=(π/30,π/30) is the ground state of the system with finite size *L*=300×300 in the periodic boundary condition for *D/J*=0.15 and *H*=0. In this magnetic structure, even under the finite magnetic field *H* perpendicular to the n_x-n_y plane, the first term of the Landau–Lifshitz–Gilbert equation Eq. (5), i.e., the torque term, vanishes at the site ***r*** for $\mathbf{q}\cdot\mathbf{r} = m\pi$ with integer *m*; particularly the magnetic moments are anti-parallel to the magnetic field stays almost static. To avoid this numerical problem, we introduce the small noise in magnetic field at the first step and later, we take a cooling period, which is typically ~1000 in units of $1/\gamma J$ with *H*=0 and α=1.0. The resulting state is the initial state (see Fig. 2(a)) for the time evolution.
